# Successful immune checkpoint inhibitor rechallenge after immune-related pericarditis: Clinical case series

**DOI:** 10.3389/fcvm.2022.964324

**Published:** 2022-08-09

**Authors:** Adrian M. Chye, Ina I. C. Nordman, Aaron L. Sverdlov

**Affiliations:** ^1^Calvary Mater Newcastle, Newcastle, NSW, Australia; ^2^Garvan Institute of Medical Research, Darlinghurst, NSW, Australia; ^3^Faculty of Medicine, St Vincent's Clinical School, University of New South Wales, Sydney, NSW, Australia; ^4^St Vincent's Hospital, Darlinghurst, NSW, Australia; ^5^School of Medicine and Public Health, University of Newcastle, Newcastle, NSW, Australia; ^6^Cardiovascular Department, John Hunter Hospital, Hunter New England Local Health District, New Lambton Heights, NSW, Australia; ^7^Newcastle Centre of Excellence in Cardio-Oncology, New Lambton Heights, NSW, Australia

**Keywords:** immune checkpoint inhibitors, immune-related adverse events, cardiotoxicity, pericarditis, lung cancer

## Abstract

Pericardial diseases secondary to immune checkpoint inhibitors (ICI) are rare. Here, we describe two cases of immune-related pericarditis caused by ICI for treatment of advanced NSCLC. Select patients can be successfully rechallenged with ICI after immune-related pericardial disease.

## Introduction

Immune checkpoint inhibitors (ICI) are a pillar of cancer therapy and have shown remarkable benefit in a range of cancer types including advanced non-small cell lung cancer (NSCLC) (1, 2). ICI are associated with a wide range of immune-related adverse events (irAE), which can affect any organ system. Cardiac irAE are rare, potentially life-threatening and include a spectrum of disorders including myocarditis, cardiomyopathy, arrhythmias and pericardial disease (3). The safety and efficacy of ICI rechallenge after cardiac irAE remains unclear. We report two rare cases (Table 1) of ICI-related pericardial disease in patients with metastatic NSCLC who were successfully rechallenged with ICI.

**Table 1:** Characteristics of patients who developed pericardial disease due to immune checkpoint inhibitors.

**Case**	**Age, yrs, sex, race**	**Cardiac irAE**	**Primary cancer and mutations**	**Other Medical history**	**CVRF**	**Cancer Stage before immunotherapy**	**Immuno-therapeutic agent**	**Onset of cardiac irAE**	**Other cancer treatment**	**Clinical presentation**	**Typical pericarditis chest pain**	**Typical ECG changes of pericarditis**	**Presence of pericardial Rub**	**Echocardiography findings: new or worsening effusion, evidence of tamponade or constriction**	**Exclusion of malignant effusion**	**Treated with high-dose steroids**	**Recovery**	**Naranjo Score**	**Recurrence of Side effect**	**Associated irAE**	**Progression of primary malignancy**	**Death/ cause of death**
1	69, male, white	Pericarditis and small pericardial effusion	Squamous cell carcinoma of the lung	COPD, Base of tongue SCC treated with radical chemoradiotherapy	Ex-smoker	IV	Nivolumab	24 weeks	Chemo- therapy and radiotherapy	Pleuritic chest pain, shortness of breath and lethargy	(+)	(-)	(-)	New small pericardial effusion, no evidence of tamponade or constriction	Tap not done, however effusion responded to steroids, colchicine	Prednsolone 50mg daily then tapered	Yes, after holding the mediciation and starting steroids	3	(-), even after drug reintroduced	Nil	(+)	(+), non-cardiac, after 5 months
2	67, female, white	Pericarditis and pericardial effusion with early tamponade, thickened pericardium	Adenocarcinoma of the lung, PD-L1 >90%, no driver mutations detected	COPD, pulmonary embolism	Hypertension (well controlled on amlodipine and valsartan)	IV	Pembroli- zumab	18 weeks	Nil	Pleuritic chest pain	(+)	(+)	(+)	New moderate pericardial effsion, early tamponade with tricspid valve velocity variation with respiration, no evidence of constriction	Tap not done, however effusion responded to steroids, colchicine	Prednsolone 50mg daily then tapered	Yes, after holding the mediciation and starting steroids	6	(+) 9 weeks after first reintroduction and 27 weeks after second reintroduction	Nil	(-)	(-)

*(–), negative; (+), positive; CK, creatine kinase; COPD, chronic obstructive pulmonary disease; CVRF, cardiovascular risk factors; ECG, electrocardiogram; irAE, immune related adverse event; IV, intravenous; PD-L1, programmed death ligand 1*.

## Learning objectives

- Clinicians should be aware of the rare and potentially life-threatening cardiac irAE related to immune checkpoint inhibitors.
- Rechallenge with immune checkpoint inhibitors following a full recovery from immune-related pericardial disease may be considered with involvement of a cardiologist or cardio-oncologist, taking into consideration the usefulness of rechallenge and predicted risk of irAE.
- Following rechallenge, patients should be monitored closely for recurrent and/or new irAE.
- If relapses occur, secondary prevention of immune-related pericardial disease can be considered with concomitant colchicine.

### Case 1

A 69-year-old male ex-smoker was diagnosed with stage IV NSCLC of squamous-cell carcinoma subtype. He initially received palliative radiotherapy to the left lower lobe bronchus obstructing primary lesion. Subsequently, he received platinum doublet chemotherapy with four cycles of carboplatin and paclitaxel, and achieved stable disease post-treatment, and was then commenced on anti-programmed death-1 (PD-1) inhibitor Nivolumab.

**Figure 1:**
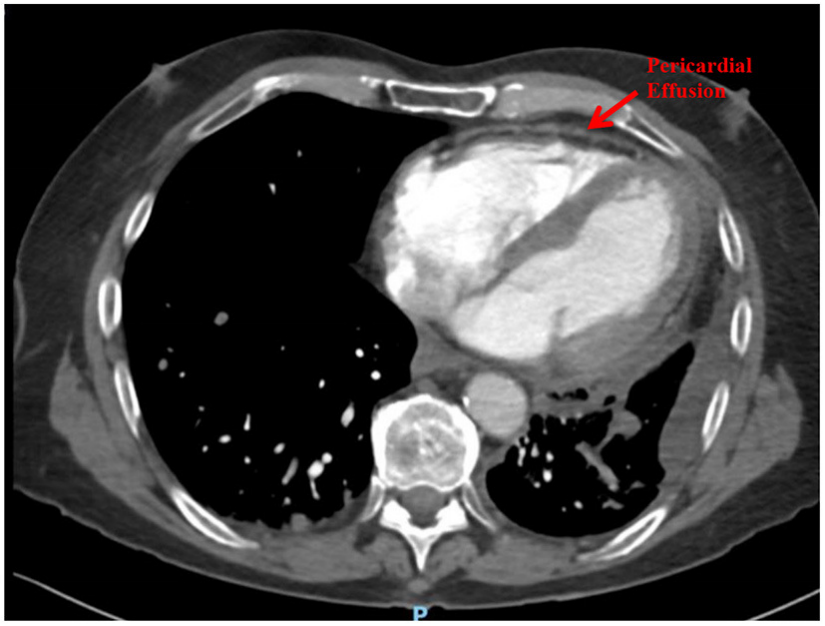
Pericardial effusion after 12 cycles of Nivolumab (Case 1).

Following 6 months (12 cycles, 2-weekly) of Nivolumab, he presented to hospital with acute pericardial chest pain, lethargy and dyspnoea, consistent with acute pericarditis. A computed tomography (CT) Pulmonary Angiogram showed a mild pericardial effusion and left pleural effusion (Figure 1). An echocardiogram demonstrated a small pericardial effusion, with no evidence of tamponade, consistent with NCI-CTCAE grade 2. He was treated with a pulse of glucocorticoids (prednisolone 50 mg daily for 1 week) and colchicine for 3 weeks. Two weeks later a repeat echocardiogram showed resolution of the pericardial effusion. He was recommenced on Nivolumab and received a further 6 cycles until disease progression, without recurrence of pericardial disease. He subsequently progressed through vinorelbine therapy and died 5 months later. There was no recurrence of the pericardial effusion at any stage.

In this case, the pericardial effusion was attributed to a cardiac irAE rather than malignant invasion/malignant effusion due to the absence of corresponding malignant disease progression prior to development of the effusion, rapid response to glucocorticoid treatment, as well as lack of recurrence of effusion when metastatic disease progressed.

A CT pulmonary angiogram demonstrated a pericardial effusion in a patient with metastatic NSCLC undergoing Nivolumab therapy.

### Case 2

A 67-year-old female was diagnosed with stage IV NSCLC of adenocarcinoma subtype, which lacked a driver mutation and programmed death ligand 1 (PD-L1) expression was >90. She achieved a partial response from frontline Pembrolizumab, an anti-PD-1 therapy.

**Figure 2:**
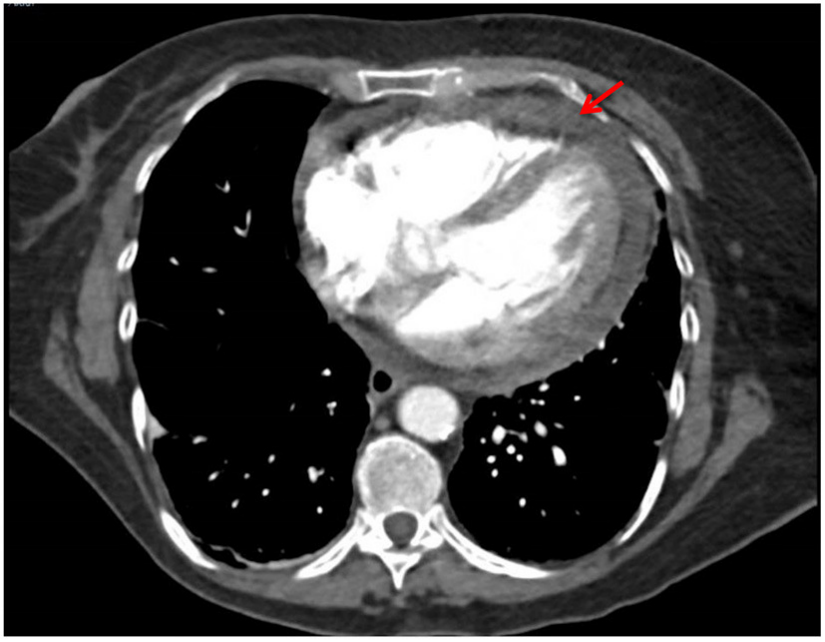
Pericardial effusion after 6 cycles of pembrolizumab (Case 2).

She presented with acute pericardial chest pain following 6 cycles (3 weekly) of Pembrolizumab. A CT pulmonary angiogram showed an interval pericardial effusion (Figure 2). An echocardiogram demonstrated a pericardial effusion with tricuspid valve velocity variation with respiration consistent with early tamponade and thickened pericardium, consistent with NCI-CTCAE grade 3. She was haemodynamically stable and did not require pericardiocentesis or surgical intervention. Pericardial disease was treated with NSAIDs, colchicine and glucocorticoids (prednisone 50 mg daily tapered over 4 weeks), with prompt resolution of symptoms. Colchicine was ceased due to diarrhea. A follow-up echocardiogram demonstrated complete resolution of the pericardial effusion. Acute pericarditis and pericardial effusion were attributed to a cardiac irAE rather than from direct malignant invasion due to the rapid response with high-dose glucocorticoid treatment, and simultaneous tumor regression in known sites of disease. She was rechallenged with pembrolizumab after multi-disciplinary consultation with the cardio-oncologist.

**Figure 3:**
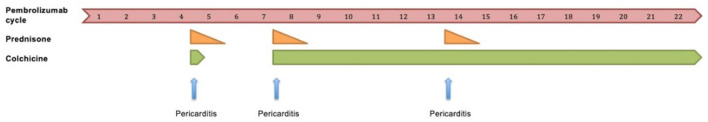
Clinical course of patient with recurrent pericarditis (Case 2).

Following cycle 9 of pembrolizumab, she represented with pericardial chest pain and was diagnosed with recurrent pericarditis. Her symptoms quickly resolved with reinstitution of NSAIDs, colchicine and glucocorticoids. Pembrolizumab was subsequently resumed concomitantly with colchicine. Following cycle 15 of pembrolizumab on concurrent colchicine therapy, a third episode of pericarditis occurred. Her symptoms again rapidly resolved with addition of NSAIDs and glucocorticoids. Pembrolizumab combined with colchicine was resumed. The patient continued to benefit from pembrolizumab with exceptional response for >18 months. The timeline of pembrolizumab treatment related to pericardial disease is shown in Figure 3.

A CT pulmonary angiogram demonstrated an interval pericardial effusion in a patient with metastatic NSCLC undergoing frontline therapy with pembrolizumab.

The development of recurrent pericarditis in relation to pembrolizumab treatment and the use of anti-inflammatory therapies including prednisone and colchicine.

## Discussion

Pericardial diseases including acute pericarditis, pericardial effusion and tamponade caused by ICI have been increasingly recognized, but very few cases have been rechallenged with ICI therapy (4–7). Overall, the incidence of pericardial disease in the setting of ICI treatment is relatively rare, in the order of 1.57 events per 100 person-years (8). We present here, two rare cases of pericardial disease caused by ICI with anti-PD-1 monotherapy who were successfully rechallenged.

The precise mechanisms by which ICI-related pericardial disease occurs is poorly understood. There is also a paucity of data evaluating the optimal management of ICI-related pericardial disease. Numerous bodies including the American Society of Clinical Oncology (ASCO) and the European Society for Medical Oncology (ESMO) have endeavored to develop standardized guidelines for managing irAE. The scope of these guidelines is restricted as they group all cardiac toxicities together, and do not address the disorder of pericardial disease specifically. ASCO guidelines recommend rapid initiation of high-dose glucocorticoids for cardiac toxicity, holding ICI and permanently discontinuing ICI if greater than grade 1 toxicity occurs (9). ESMO suggest treating cardiac toxicity with high-dose glucocorticoids and escalation to other immunosuppressive drugs if not responsive to glucocorticoids (10).

ICI-related pericardial disease is treated with high-dose glucocorticoids (prednisolone 1–2 mg/kg), urgent pericardiocentesis if haemodynamic compromise is present, and permanent discontinuation of ICI. The safety, efficacy, and appropriateness of rechallenging with ICI following ICI-related pericardial disease is a key question that is yet to be answered. In routine clinical practice, ICI is permanently discontinued in patients who have developed cardiac irAE. We propose that patients with ICI-related pericardial disease with resolution or near resolution of irAE, ICI re-challenge can be considered on a case-by-case basis with multi-disciplinary expertise. The multi-disciplinary team should consider the potential benefit of a rechallenge, patient comorbidities, patient preference and predicted risk of new and recurrent irAE. Patients should be closely monitored for recurrent or new irAE if ICI is indeed resumed.

Furthermore, Case 2 demonstrates that if relapses of ICI-related pericarditis do occur, it can be managed with temporary discontinuation of ICI and short courses of glucocorticoids. Recurrent pericarditis in this case was immunosuppression-sensitive and each relapse was followed by a full recovery. Following cardio-oncology consultation, discussion with the patient and careful consideration of the risk-benefit ratio of resuming treatment, ICI was reinstituted. Secondary prevention for ICI resumption with long-term concomitant colchicine was used as a strategy to reduce the risk of recurrent pericarditis.

A systematic review of case reports and series, which included 20 publications with a total of 28 cases of ICI-associated pericardial disease, suggested that majority of cases are severe, and the re-challenge was only done in minority (7 out of 28 patients) in the absence of further pericardial effusion (11). Our case series demonstrates that re-challenge is possible even in the case of recurrent pericarditis/pericardial effusion, provided it is not severe. This may potentially help avoid unnecessary discontinuation of life-saving ICI therapy in patients with mild pericardial disease, even if it recurs.

One limitation of our case series was that pericardial effusion cytology or pericardial biopsy were not performed on either patient, due to non-life-threatening nature of the pericardial effusions and rapid response to treatment.

## Conclusion

Clinicians across disciplines should be aware of the rare but potentially life-threatening complication of ICI-related pericardial disease to enable prompt diagnosis and treatment. Rechallenge with ICI following resolution of pericardial disease on prior ICI treatment may be considered, but should be balanced with the usefulness of rechallenge, patient comorbidities and the risk of recurrent irAE. If relapses occur, secondary prevention of ICI-related pericardial disease can be considered with concomitant colchicine. Questions remain regarding the pathogenesis of irAE, optimal management of cardiac irAE and in what circumstances ICI may be resumed after irAE. Further prospective clinical trials are needed to address these gaps in our knowledge.

## Data availability statement

The original contributions presented in the study are included in the article/supplementary material, further inquiries can be directed to the corresponding author.

## Ethics statement

Written informed consent was obtained from the individual(s) for the publication of any potentially identifiable images or data included in this article. Written informed consent was obtained from the participant/s for the publication of this case report.

## Author contributions

AC wrote the manuscript in consultation with IN and AS. All authors were involved in the case management, contributed to the article, and approved the submitted version.

## Funding

AS is supported by a Future Leader Fellowship (Award Reference No. 106025) from the National Heart Foundation of Australia. This work, in part, was supported by the Cardiovascular Medicine Department at the Hunter New England Local Health Service, Australia. The funder had no role in the current work.

## Conflict of interest

AS has received speaker honorarium/advisory board/consultancy fees from Bayer, Novartis, BMS, AstraZeneca, and Boehringer Ingelheim and research grants from BMS, Roche, Vifor, Biotronik, and RaceOncology.

The remaining authors declare that the research was conducted in the absence of any commercial or financial relationships that could be construed as a potential conflict of interest.

## Publisher's note

All claims expressed in this article are solely those of the authors and do not necessarily represent those of their affiliated organizations, or those of the publisher, the editors and the reviewers. Any product that may be evaluated in this article, or claim that may be made by its manufacturer, is not guaranteed or endorsed by the publisher.

## Abbreviations

CT: computed tomography
PD-1: programmed cell death protein 1
PD-L1: programmed cell death ligand 1
ICI: immune checkpoint inhibitor
IrAE: immune-related adverse event
NSCLC: non-small cell lung cancer.
